# Development and Preliminary Validation of the “Teacher of Physical Education Burnout Inventory” (TPEBI) in Arabic Language: Insights for Sports and Occupational Psychology

**DOI:** 10.3389/fpsyg.2019.00456

**Published:** 2019-04-09

**Authors:** Nasr Chalghaf, Noomen Guelmami, Maamer Slimani, Giovanni Del Puente, Tania Simona Re, Riccardo Zerbetto, Juan José Maldonado Briegas, Ottavia Guglielmi, Sergio Garbarino, Fairouz Azaiez, Nicola Luigi Bragazzi

**Affiliations:** ^1^Postgraduate School of Public Health, Department of Health Sciences (DISSAL), University of Genoa, Genoa, Italy; ^2^Department of Neuroscience, Rehabilitation, Ophthalmology, Genetics, Maternal and Child Health (DINOGMI), University of Genoa, Genoa, Italy; ^3^Higher Institute of Sport and Physical Education, University of Sfax, Sfax, Tunisia; ^4^Studies Group of Development and Social Environment, Faculty of Letters and Human Sciences of Sfax, University of Sfax, Sfax, Tunisia; ^5^Higher Institute of Sport and Physical Education of Kef, Kef, Tunisia; ^6^UNESCO Chair “Health Anthropology, Biosphere and Healing Systems”, University of Genoa, Genoa, Italy; ^7^Department of Psychology and Sociology of Education, University of Extremadura, Badajoz, Spain; ^8^Centro Studi Terapia della Gestalt, Milan, Italy

**Keywords:** development and validation of a questionnaire, Arabic language, sports psychology, occupational psychology, work and well-being, burnout, teachers

## Abstract

**Background:** Burnout is an inappropriate response to chronic work stress, leading to emotional exhaustion (EE), depersonalization (D), and low personal accomplishment (PA). Burnout can affect workers in the helping professions. To quantitatively assess the burnout level among teachers, Maslach has adapted the “Maslach Burnout Inventory” (MBI) to the educational environment (the so-called MBI Educators Survey version or MBI-ES). Among teachers, sports and physical education teachers may suffer from burnout due to high workload.

**Aims:** No reliable psychometric tool in Arabic language exists that can be used to measure the burnout level among sports and physical education teachers. The objective of the present study was to develop a burnout measurement scale according to the Maslach’s three-dimensional theoretical model for physical education teachers in Tunisia and to test its factor structure, in terms of internal consistency/reliability, predictive validity, and sensitivity.

**Methods:** A total of 525 Tunisian teachers teaching in secondary schools from different Tunisian governorates volunteered to participate in this study. The sample comprised of 285 males (54.3%) and of 240 females (45.7%). More in detail, 327 were teachers of primary school of physical education (62.3%) and 198 teachers of secondary school (37.7%). Teachers were administered both the *ad hoc* developed “Teacher of Physical Education Burnout Inventory” (TPEBI) and the MBI-ES. Both exploratory [principal component analysis (PCA)] and confirmatory factor analyses (CFAs) were performed.

**Results:** The Cronbach’s alpha coefficients were excellent (0.93, 0.94, and 0.91 for EE, D, and PA, respectively). The correlation matrix indicated significant correlations between the TPEBI and MBI-ED dimensions. However, CFA fit indices were not completely satisfactory.

**Conclusion:** Given the good PCA factor loadings, the correlation matrix, the sensitivity analysis, and the excellent internal consistency, it can be concluded that the TPEBI is a reliable psychometric tool that can be used to quantitatively assess the burnout level among teachers of physical education in the Arabic-speaking world. However, considering the CFA fit indices, further modifications to fully support the model are warranted.

## Background

Initially observed inside a detoxification clinics and introduced by Freudenberger (1974), the concept of burnout has attracted considerable attention in the recent decades. Burnout can be described as an inappropriate response to chronic work stress, which leads to emotional exhaustion (EE), depersonalization (D), and low personal accomplishment (PA). According to Maslach, burnout is a syndrome of EE, D, and loss of self-efficacy, which may affect those individuals working with and caring for other humans, as a response to the emerging chronic emotional burden (Cherniss, 1980; Maslach, 1982; Maslach and Jackson, 1985, 1986; Golembiewski et al., 1986; Maslach and Schaufeli, 1993; Maslach et al., 2001).

Burnout, as such, can affect workers in the helping professions, like nurses, doctors, social workers, and teachers. These workers may experience high emotional stress due to frequent and intensive interactions with others. For instance, in the field of education, many studies have noted that the teaching profession is becoming more and more stressful, demanding and challenging, and that teacher burnout is an emerging, international psychosocial concern (Koustelios, 2001; Tsigilis et al., 2004; Koustelios and Tsigilis, 2005; Montgomery and Rupp, 2005; Hakanen et al., 2006; Stoeber and Rennert, 2008; Chang, 2009; Skaalvik and Skaalvik, 2011; Liu and Onwuegbuzie, 2012).

According to a meta-analysis, which has pooled together 2,527 correlational effect sizes from 65 studies, teachers are particularly vulnerable to stress and may suffer from ineffective mediating coping mechanisms over a long period of time (Montgomery and Rupp, 2005). Teacher burnout may have devastating consequences both for the mental health of teachers and students and may affect and compromise the quality of education (De Stasio et al., 2017; Benevene et al., 2018). Moreover, the higher the level of teacher’s breakdown, the more likely the consequence of stress and burnout in the teaching profession (Koustelios, 2001; Tsigilis et al., 2004; Koustelios and Tsigilis, 2005; Montgomery and Rupp, 2005; Hakanen et al., 2006; Stoeber and Rennert, 2008; Chang, 2009; Skaalvik and Skaalvik, 2011; Liu and Onwuegbuzie, 2012).

With respect to the general population, teachers tend to have a higher level of burnout and, in particular, of EE, which can negatively impact on the learning process of students (Montgomery and Rupp, 2005).

Physical education teachers are particularly exposed to burnout. Indeed, physical education classes take place mainly outside the classrooms, which causes more problems to maintain discipline than in the classroom and requires constant vigilance for ensuring students’ safety (Tsigilis et al., 2011). In addition, physical education teachers are often obliged to teach in particular situations and conditions, dealing with students who may be particularly turbulent, noisy, and intractable (Brouwers et al., 2011). At the end of the day, as such, physical education teachers may be physically and mentally exhausted (Brouwers et al., 2011).

In order to quantitatively assess the burnout level among teachers, Maslach has adapted the “Maslach Burnout Inventory” (MBI) to the educational environment (the so-called MBI Educators Survey version or MBI-ES). Many researchers have utilized this tool to investigate the phenomenon of burnout in various educational settings (primary, intermediate and secondary schools) in different cultural contexts such as the United States (Boles et al., 2000), Canada (Byrne, 1991), Holland (Schaufeli et al., 1994), Greece (Kantas and Vassilaki, 1996; Antoniou et al., 2006),Cyprus (Kokkinos, 2006), and Sweden (Arvidsson et al., 2016). The results of these investigations seem to suggest that teachers working in European countries suffer from lower levels of burnout than their colleagues in the United States or Canada, putatively due to cultural, societal, and organizational factors. Van Horn et al. (1997) have managed to replicate such finding, showing that Dutch teachers had a lower level of EE and D when compared to their Canadian counterparts. This result is of crucial importance, when validating the psychometric tool in another language and adapting to another culture/context.

Among teachers, sports and physical education teachers may suffer from burnout due to high workload. For instance, a study carried out in Pelotas, in the southern state of Rio Grande do Sul (Brazil), found that 60.6% of teachers experienced high EE, 22.3% high D, and 34.0% low PA. According to the scores, 8.5% of teachers suffered from burnout syndrome (Sinott et al., 2014).

## Aims

To the best of our knowledge, there is no psychometric tool in Arabic language that can be used to measure the burnout level among sports and physical education teachers. Therefore, the objective of the present study was to develop burnout measurement scale according to the Maslach’s three-dimensional theoretical model for physical education teachers in Tunisia and to test its factor structure, in terms of internal consistency/reliability, predictive validity, and sensitivity.

## Materials and Methods

### Psychometrics Instruments

#### Development of the TPEBI

The “Teacher of Physical Education Burnout Inventory” (TPEBI) was devised based on the three dimensions of the Maslach’s theoretical model (EE, D and PA).

The questionnaire was developed through different steps and phases: namely, an initial broad and comprehensive literature review on the topic (including, for instance, Chang, 2009; Skaalvik and Skaalvik, 2011; Liu and Onwuegbuzie, 2012). At this step, we could identify the main aspects that characterize the dimensions of burnout. Moreover, we made efforts to integrate the specific characteristics of the population under study into the questions. When drafting the items of the questionnaire, we selected a vocabulary that was clearly comprehensible and unambiguous. In the subsequent step, we performed a search of similar questionnaires in other languages and tested in other countries/settings (such as Montgomery and Rupp, 2005; Skaalvik and Skaalvik, 2011; Liu and Onwuegbuzie, 2012). In the next step, a focus group was arranged with experts in the Arab language, in sports and physical education, and in human and applied sciences. Then, several corrections made it possible to reformulate the questions that appeared to be unclear. This allowed the improvement of the tool. Finally, a pilot study was carried out, testing the preliminary properties and the readability of the questionnaire. This test confirmed the validity of the tool.

#### The MBI-ES

The MBI-ES is a psychometric instrument originally developed by Maslach et al. (1996), explicitly designed to quantitatively assess the burnout level among teachers. The tool is a 22-item questionnaire divided into three dimensions: seven items measuring EE, six items dealing with D, and nine items exploring PA. The scores are obtained on a six-point Likert scale and each dimension is calculated by computing the sum of the items. Several studies investigating both the original version and its cross-cultural adapted versions have confirmed the psychometric robustness of the tool, in terms of internal consistency and factor structure. In particular, two studies have confirmed the validity and reliability of the MBI-ES. Factor analysis studies by Iwanicki and Schwab (1981) and Gold (1984) supported, indeed, the MBI-ES 3-factor structure. Regarding reliability, Iwanicki and Schwab (1981) have reported Cronbach’s alpha estimates of 0.90 for EE, 0.76 for D, and 0.76 for PA, while Gold (1984) reported estimates of 0.88, 0.74, and 0.72, respectively.

### Ethics Statement

The study protocol of the present investigation received ethical clearance from the UNESCO Chair “Health Anthropology Biosphere and Healing Systems,” University of Genoa, Genoa (Italy), the Higher Institute of Sport and Physical Education of Sfax, Sfax (Tunisia), the Faculty of Letters and Human Sciences of Sfax, Sfax (Tunisia), and the Higher Institute of Sport and Physical Education of Kef, Kef (Tunisia). The project was approved by the Ethical Committee of the University of Sfax, Sfax, Tunisia.

All participants to the present study provided written, informed consent. Teachers were extensively informed about the purposes and procedure of the study, and were advised that the results would be made available to them upon completion of the study only in aggregate form, with no possibility to trace back to the single teacher’s scores, thus ensuring anonymity and preserving the privacy of each participant.

The present investigation was carried out in accordance with the ethical principles of the 1964 Helsinki declaration and its subsequent amendments.

### Procedure

Teachers who agreed to participate in the study were instructed how to proceed and complete the survey procedures required by the present study. Following the agreement of the secondary school principals, copies of the TPEBI and of the MBI-ES were simultaneously distributed to teachers at their work sites in off-peak hours. The entire procedure of questionnaires administration took over 2 months. A proper time (approximately 30 min) was ensured to each participant in order to answer the questionnaire thoroughly.

### Statistical Analysis

#### Descriptive Analysis

Before commencing any statistical analysis, data were visually inspected for potential outliers. Normality of data distribution was checked using the Pearson-D’Agostino *omnibus* test. Questionnaires scores were also checked for skewness and kurtosis, computing the Mardia’s multivariate skewness and kurtosis statistics.

#### Internal Consistency/Reliability

The internal consistency of the instrument was examined computing the Cronbach’s alpha coefficient for all the three dimensions of the inventory. More in detail, in order to properly interpret the alpha coefficient, the following rule of thumb was used (Nunnally, 1978; George and Mallery, 2011): the coefficient was deemed excellent if the estimate was >0.90, whereas it was judged good in the range 0.80–0.90, acceptable in the range 0.70–0.80, questionable or adequate in the range 0.60–0.70, poor in the range 0.50–0.60, and unacceptable if <0.50.

#### Inferential Statistics – Sensitivity Analysis

The sensitivity of the instrument was tested by performing a univariate analysis of variance (ANOVA), examining the impact of teachers’ grade, gender, age, and their interaction effects on the TPEBI three dimensions scores.

#### Predictive Validity

Predictive validity was tested computing the Pearson’s correlation between the dimensions of the *ad hoc* devised psychometric instrument (the TPEBI) and those of the MBI-ES. More in detail, the strength of correlation was measured using the rule of thumb described by Hinkle et al. (2003): the correlation was deemed negligible with the *r* coefficient in the range 0.00–0.30, low with *r* in the range 0.30–0.50, moderate with *r* in the range 0.50–0.70, high with *r* in the range 0.70–0.90, and, finally, very high with *r* in the range 0.90–1.00.

#### Principal Component Analysis (PCA)

The factor structure was initially investigated by conducting a principal component analysis (PCA) and a *varimax* rotation with Kaiser Normalization. More in detail, *varimax* rotation was chosen in that this approach enables to minimize factor complexity while, at the same time, maximes the variance of factor loadings (Tabachnick and Fidell, 2013).

Before proceeding with the PCA, the Kaiser–Meyer–Olkin (KMO) measure was computed in order to assess the sampling adequacy. Ideally, the KMO should be greater than 0.60. Once verified the sampling adequacy, a PCA iterative strategy was adopted in the present investigation. Different runs were carried out. First, an exploratory/preliminary run was performed on the 24 items of the questionnaire without conducting any rotation, in order: (i) to check if this approach could be deemed an appropriate technique for the matrix by examining whether the correlations among items were satisfactory (that is to say, yielding values > 0.30) and (ii) to control for the factorability of the correlation matrix utilizing the Bartlett’s test of sphericity. In cases of statistical significance, this test enables scholars to reject the null hypothesis (that is to say the correlations in the correlation matrix are zero and the matrix is an identity matrix).

The likely number of factors was determined by: (i) computing the number of factors with eigenvalues greater than 1 (Field, 2009; Tabachnick and Fidell, 2013) and (ii) visually inspecting the Cattell’s scree-plot. After checking the factor loadings, items were deleted in cases of unsatisfactory loading (that is to say, values less than 0.45). Moreover, items were suppressed if their factor loading conflicted with a sound theoretical explanation (Field, 2009; Tabachnick and Fidell, 2013). In the present case, no items were deleted, since all were retained.

Different runs with *varimax* rotation were, therefore, carried out in an iterative fashion, as previously explained, until a satisfactory, clearly interpretable solution was finally obtained. Cases of cross-loading were interpreted according to salience and explained variance, with theoretical considerations also being taken into account (Field, 2009; Tabachnick and Fidell, 2013).

#### Confirmatory Factor Analysis (CFA)

Then the model was tested by confirmatory factor analysis (CFA). As suggested and recommended by many scholars, a wide range of fit indices was calculated and reported, namely: (i) discrepancy indices [including the chi-squared and the Steiger–Lind’s root-mean-square error of approximation (RMSEA)], (ii) tests comparing the target model with the null model [like the Bentler–Bonett’s normed fit index (NFI), the Bentler–Bonett’s not normed fit index (NNFI), known also as the Tucker–Lewis’ index (TLI), the Bentler’s comparative fit index (CFI), and the James–Mulaik–Brett’s parsimony goodness-of-fit index (PGFI)], and (iii) information theory goodness-of-fit measures [the Joreskog’s goodness-of-fit index (GFI), and the Joreskog’s adjusted GFI (AGFI)].Concerning the cut-off and threshold values for discrepancy indices, the *p-value* associated with the chi-squared test should exceed 0.05 (that is to say, it should not be statistically significant). Further, the chi-squared divided by the degrees of freedom (df) value, should ideally be less than 2.0. As far as the RMSEA is concerned, values higher than 0.10 indicate poor fitting models (Steiger, 2000). Concerning the cut-off and threshold values for tests that compare the target model with the null model, NFI should exceed 0.90 according to Byrne (1994) or 0.95 according to Schumacker and Lomax (2004). NNFI/TLI should be above 0.95 according to Hu and Bentler (1995). PGFI is derived from NFI, correcting and compensating for model parsimony. CFI should exceed 0.95 (Bentler, 1990; Hu and Bentler, 1999) or 0.90 according to other scholars. Finally, regarding the cut-off and threshold values for information theory goodness-of-fit measures, GFI value should be higher than 0.90 (Byrne, 1994).

#### Statistical Software

All statistical analyses were carried out using the commercial software “Statistical Package for the Social Sciences” (IBM SPSS software for Windows, version 21.0, IBM Corp., Armonk, NY, United States; released 2012) whereas the CFA was performed by utilizing the commercial software “Analysis of a moment structures” (Amos software for Windows, version 21.0, IBM, SPSS, Chicago, United States; Arbuckle, 2012a,b).

For all statistical analyses, figures with *p*-value less than 0.05 were considered statistically significant.

## Results

### Univariate and Multivariate Normality

Concerning univariate normality, scores for all the items of the TPEBI items had univariate normal distributions with acceptable values of skewness and kurtosis.

As far as multivariate normality is concerned, the Mardia coefficients showed evidence of multivariate non-normality in the data (multivariate kurtosis 782.35, *z* = 23.69, *p* < 0.001, and multivariate skewness 61.85, *z* = 29.95 *p* < 0.001).

### Development of the Psychometric Instrument: The TPEBI

Based on the different steps and phases mentioned in Section “Materials and Methods,” the *ad hoc* devised psychometric instrument is made up of 24 items (eight items for each dimension, EE, D, and PA), and the scores of the dimensions are obtained by averaging the items scores. The answers are coded on a seven-point Likert scale. The 24 items of the TPEBI are reported in Table 1.

**Table 1:** Items of the *ad hoc* devised psychometric tool to quantitatively assess the burnout level among the teachers of physical education in the Arabic-speaking world, the “Teacher Physical Education Burnout Inventory” (TPEBI).

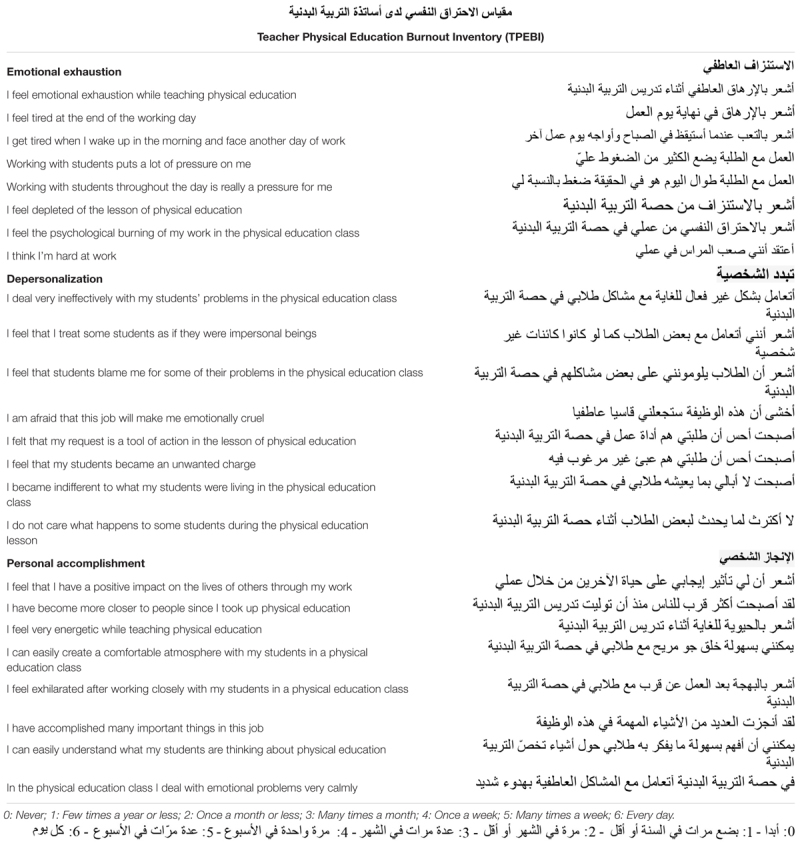

### Descriptive Analysis

At the collection and analysis of the questionnaires, there were no missing data. A total of 525 Tunisian teachers teaching in secondary schools from different Tunisian governorates volunteered to participate in this study. The sample comprised of 285 males (54.3%) and of 240 females (45.7%). More in detail, 327 were teachers of primary school of physical education (62.3%) and 198 teachers of secondary school (37.7%). Based on age distribution, the subjects were categorized into four age groups: namely, (i) age < 39 years, *n* = 113 (21.5%), (ii) age between 39 and 44 years, *n* = 220 (41.9%), (iii) age between 44 and 49 years, *n* = 105 (20.0%), and (iv) age > 49 years, *n* = 87 (16.6%).

### Principal Component Analysis (PCA)

Based on the eigenvalues and the inspection of the Cattell’s scree-plot, PCA revealed a three-factor structure, accounting for 63.68% (Tables 2, 3). Factor loadings ranged from 0.73 to 0.89 for EE, from 0.76 to 0.82 for D, and from 0.69 to 0.84 for PA.

**Table 2:** Descriptive statistics reporting the scores of the “Teacher of Physical Education Burnout Inventory” (TPEBI) for each dimension found performing the principal component analysis (PCA).

Grade	Gender	Age	First dimension		Second dimension		Third dimension	
					
			Mean	*SD*	Mean	*SD*	Mean	*SD*
TPS	M	1	4.25	1.25	3.72	1.83	3.91	1.36
		2	3.92	1.46	3.38	1.31	3.67	1.54
		3	4.15	1.61	3.82	2.04	4.05	1.60
		4	4.49	1.55	3.23	1.54	4.15	1.51
	F	1	3.75	1.53	3.36	1.39	3.64	1.47
		2	3.28	1.31	3.65	1.44	3.27	1.26
		3	4.15	1.56	2.95	1.44	3.51	1.50
		4	4.04	1.49	3.62	1.54	3.98	1.14
TSS	M	1	2.80	1.10	2.77	1.92	4.23	1.25
		2	3.23	1.49	3.34	1.12	2.71	1.12
		3	3.21	0.86	3.87	1.54	3.47	1.58
		4	3.32	1.40	3.06	1.52	3.28	0.95
	F	1	3.29	1.43	2.69	1.54	3.12	1.32
		2	3.25	1.51	3.30	1.26	2.83	1.20
		3	3.48	1.24	3.53	1.38	3.44	1.63
		4	2.98	1.50	2.39	1.08	3.03	1.16


*F (female); M (male); SD (standard deviation); TPS (teacher of primary school): TSS (teacher of secondary school). Scores are broken down by teachers’ grade, gender, and age group.*

**Table 3:** Factor loadings for the three-factor solution of the “Teacher of Physical Education Burnout Inventory” (TPEBI) questionnaire.

Item	Emotional exhaustion	Depersonalization	Personal achievement
I11	0.89		
I12	0.85		
I9	0.84		
I14	0.84		
I13	0.83		
I10	0.82		
I15	0.81		
I16	0.73		
I1		0.82	
I5		0.82	
I6		0.81	
I7		0.81	
I3		0.80	
I8		0.79	
I2		0.78	
I4		0.76	
I22			0.84
I23			0.82
I19			0.76
I21			0.76
I18			0.74
I20			0.73
I24			0.72
I17			0.69


*Exploratory factor analysis was performed with *varimax* rotation and Kaiser normalization.*

### Internal Consistency/Reliability

The Cronbach’s alpha coefficients were 0.93, 0.94, and 0.91 for EE, D, and PA, respectively. As such, the internal consistency/reliability of the *ad hoc* devised psychometric tool was found to be excellent.

### Inferential Statistics – Sensitivity Analysis

The results of the ANOVA are reported in Table 4. Grade significantly impacted on all the three dimensions of the TPEBI. Gender and age significantly impacted on the PA dimension of the TPEBI. Concerning the interaction effects, no significant influence could be found except for the interaction grade × age, significantly impacting on the D dimension of the TPEBI.

**Table 4:** Impact on grade, gender, age, and their interaction effects on the “Teacher of Physical Education Burnout Inventory” (TPEBI) dimension and total scores.

Variable	F		
	
	Emotional exhaustion	Depersonalization	Personal achievement
Grade	30.03^∗∗^	5.29^∗^	12.87^∗∗^
Gender	0.95	1.98	5.50^∗^
Age	1.36	2.04	5.89^∗∗^
Grade × gender	2.98	0.22	0.01
Grade × age	1.67	2.75^∗^	1.64
Gender × age	0.71	1.26	0.93
Grade × gender × age	0.41	1.11	1.61


*^∗^(significant at 0.5 level); ^∗∗^(significant at 0.01 level).*

### Predictive Validity

The correlation matrix showed a number of statistically significant correlations between the TPEBI and the MBI-ES dimensions. Correlations are shown in Table 5. More in detail, concerning the TPEBI, correlation between PA and EE dimensions yielded a value of 0.19 (negligible, even though significant at 0.001 level). EE dimension of the TPEBI correlated with the EE (*r* = 0.71, high correlation, significant at 0.001 level), with the D (*r* = 0.18, negligible correlation, even though significant at 0.001 level) and with the PA (*r* = 0.17, negligible correlation, even though significant at 0.001 level) dimensions of the MBI-ES. The D dimension of the TPEBI correlated with the EE (*r* = 0.15, negligible correlation, even though significant at 0.001 level), and with the D (*r* = 0.65, moderate correlation, significant at 0.001 level) dimensions of the MBI-ES. Finally, the PA dimension of the TPEBI correlated with the EE (*r* = 0.20, negligible correlation, even though significant at 0.001 level), and with the PA (*r* = 0.71, high correlation, significant at 0.001 level) dimensions of the MBI-ES.

**Table 5:** Correlation between the “Teacher of Physical Education Burnout Inventory” (TPEBI) and the “Maslach Burnout Inventory – Educators Survey” (MBI-ES) dimensions.

Correlations	
		**EE TPEBI**	**DTPEBI**	**PA TPEBI**	**EE MBI-ES**	**D MBI-ES**	**PA MBI-ES**
EE TPEBI	Pearson correlation	1.00	0.02	0.19^∗∗∗^	0.71^∗∗∗^	0.18^∗∗∗^	0.17^∗∗∗^
	Sig. (two-tailed)		0.620	0.000	0.000	0.000	0.000
D TPEBI	Pearson correlation	0.02	1.00	0.04	0.15^∗∗∗^	0.65^∗∗∗^	0.01
	Sig. (two-tailed)	0.620		0.318	0.000	0.000	0.814
PA TPEBI	Pearson correlation	0.19^∗∗∗^	0.04	1.00	0.20^∗∗∗^	-0.03	0.71^∗∗∗^
	Sig. (two-tailed)	0.000	0.318		0.000	0.475	0.000
EE MBI-ES	Pearson correlation	0.71^∗∗∗^	0.15^∗^	0.20^∗∗∗^	1.00	0.20^∗∗∗^	0.14^∗∗^
	Sig. (two-tailed)	0.000	0.000	0.000		0.000	0.001
D MBI-ES	Pearson correlation	0.18^∗∗∗^	0.65^∗^	-0.03	0.20^∗∗∗^	1.00	-0.02
	Sig. (two-tailed)	0.000	0.000	0.475	0.000		0.680
PA MBI-ES	Pearson correlation	0.17^∗∗∗^	0.01	0.71^∗∗∗^	0.14^∗∗^	-0.02	1.00
	Sig. (two-tailed)	0.000	0.814	0.000	0.001	0.680	


^∗^(significant at 0.05 level); ^∗∗^(significant at 0.01 level); ^∗∗∗^(significant at 0.001 level); D (depersonalization); EE (emotional exhaustion); PA (personal achievement).

### Confirmatory Factor Analysis (CFA)

**FIGURE 1:**
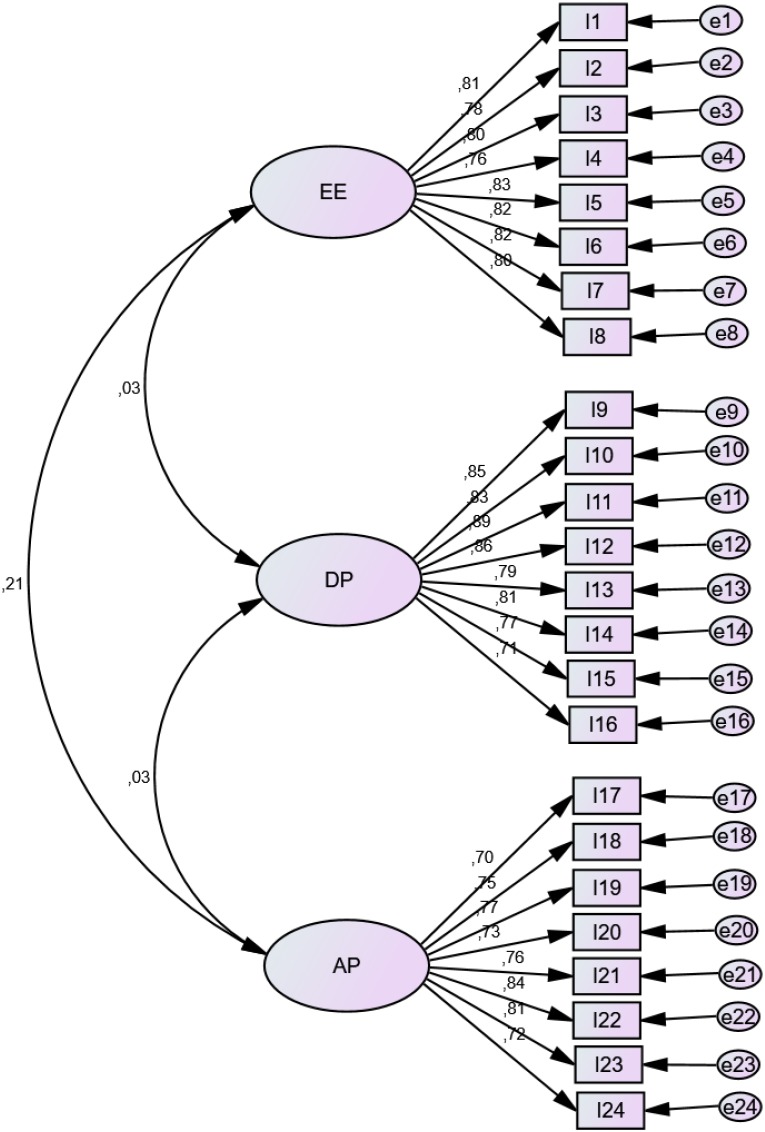
Findings of the confirmatory factor analysis (CFA) for the “Teacher of Physical Education Burnout Inventory” (TPEBI).

Concerning the CFA indices, the chi-squared yielded a value of 1592.69 (df = 249, chi-squared/df = 6.40). GFI and AGFI resulted 0.74 and 0.69, respectively. PGFI was 0.61, whereas TLI 0. 85 and CFI 0.86. RMSEA was 0.1. The findings of the CFA are pictorially shown in Figure 1.

## Discussion

The objective of the present study was to construct and to test the factor structure, internal consistency/reliability, predictive validity, and sensitivity of an *ad hoc* burnout measurement scale for the Arabic-speaking world, devised according to the Maslach’s three-dimensional theoretical model. Once developed, the 24-item tool was validated in a representative sample of physical education teachers in Tunisia, using both PCA and CFA. PCA factor loadings were good and the CFA fit indices satisfactory. The internal consistency/reliability of the three dimensions was found to be excellent. Furthermore, the instrument showed a number of statistically significant correlations with an already validated tool, the MBI-ES for teachers. Moreover, it was able to differentiate the burnout level according to the grade of the physical education teachers at the level of all the three dimensions, and, even though only partially, also according to age and gender of the teachers.

Generally, our findings are consistent with the existing scholarly literature on the topic. For instance, Kokkinos (2006) tested the three-factor burnout model in a population of physical education teachers in Cyprus using both PCA and CFA techniques. The results proved the psychometric robustness and soundness of the instrument in terms of factor loadings and, to a less extent, of CFA fit indices. The internal consistency/reliability was satisfactory, with Cronbach’s alpha coefficients of 0.85 for EE, 0.63 for D, and 0.79 for PA. Female teachers seemed to be more emotionally exhausted than their male counterparts. Primary school teachers were more affected by EE, but D was higher among secondary school teachers. Overall, the results suggested that the Greek MBI-ES represented a valid and reliable adaptation of the original instrument, which can be used with confidence for in field investigations aimed to measure burnout.

In a recent cross-sectional study by Spittle et al. (2015), burnout was investigated in a sample of 49 high school physical education teachers, aged 25–63 years, stratifying the analysis by age and gender. Authors found that burnout scores did not significantly differ by gender, whereas age impacted on the PA dimension, with younger teachers reporting lower levels of PA, therefore indicating greater levels of burnout. No significant interaction between gender and age was found for the aspects of EE or PA, but the effect was, instead, vital for the D dimension; younger male teachers reported, indeed, higher scores (moderate level) for D than older male teachers (low level), while younger and older female teachers reported comparable scores. These results indicated that the most inexperienced teachers in physical education tended to suffer more from burnout, which seems to be particularly true for young male teachers.

In another comparative study of Tsigilis et al. (2011) between physical education teachers at primary and secondary schools, it was shown that physical education teachers working in primary schools had significantly higher scores of burnout, namely, EE, compared to their secondary school colleagues. In addition, the strength of association between the three components of burnout was greater among primary school physical educators than in secondary school ones (Tsigilis et al., 2011).

In another investigation, Arvidsson et al. (2016) examined the prevalence rate of burnout among Swedish teachers, using a cross-culturally adapted version of the MBI-ES. Results reported low values in all the three dimensions of the inventory and there was no association between gender and increasing levels of burnout among teachers.

Studies addressing the relationship between socio-demographic data and teacher burnout have consistently shown that some baseline factors can predict low but statistically significant variance in, at least some, subscales/dimensions of the burnout measurement questionnaire. Generally, age has been shown to be a significant predictor of EE, with younger teachers tending to score higher than older teachers. Considering gender, male teachers have been found to tend to have a higher score than female teachers on the D scale.

This finding is consistent with research conducted among other helping professions (Maslach and Jackson, 1985). Teachers who work with secondary school students tend to have a lower level of PA/personal success than their counterparts in primary school. Secondary school teachers also feel more depersonalized toward students than primary or junior teachers (Maslach and Jackson, 1985).

Grade represents an important variable that can be used to predict burnout levels. Differentiating between primary and secondary school teachers is essential in the field of burnout. Indeed, the survey by Kantas and Vassilaki (1996) on Greeks teachers showed significant differences between secondary and primary teachers. Furthermore, Kantas and Vassilaki (1996) showed for secondary school physical education teachers significantly lower scores for EE and D and significantly higher scores for PA.

Finally, teacher burnout syndrome seems to fluctuate depending on the cultural context and the educational system in which it is studied. For example, previous research has shown that North American teachers are more likely to be vulnerable to burnout than the Europeans (Koustelios, 2001).

### Strengths, Limitations, and Future Prospects

The major strength of the present investigation lays in its uniqueness and usefulness. The validated instrument is, indeed, expected to be concretely useful for improving the health and well-being of Arab speaking teachers of physical education. A further strength is given by the methodological rigor of the research: being the development and validation of an instrument, it follows the rules which are nowadays considered a standard in this kind of studies. An in-depth analysis of the psychometric properties of the questionnaire, using both PCA and CFA, has been carried out.

Among the limitations, even though in general from a psychometric standpoint the instrument is sound, based on the CFA findings, some modifications could be still required in order to further fully support the factor structure. Future studies should be conducted on other samples to replicate the current results.

Furthermore, given that the Arabic speaking world is quite vast and culturally various and that the current validation analysis was performed only in Tunisia, further studies in other Arabic speaking countries are warranted in order to strengthen the findings.

## Conclusion

Our study aimed to develop and to test the factor structure, internal consistency/reliability, predictive validity, and sensitivity of a burnout measurement scale. Given the good PCA factor loadings, the correlation matrix, the sensitivity analysis, and the excellent internal consistency, it can be concluded that the TPEBI is a reliable psychometric tool that can be used to quantitatively assess the burnout level among teachers of physical education in the Arabic-speaking world. However, considering the CFA fit indices, some modifications are still required and further studies with higher samples are warranted in order to fully support the factor model.

## Author Contributions

NC, FA, and NB conceived the experiment and drafted the manuscript. NC performed the experiment. NC, NG, FA, and NB collected and analyzed the data. SM, GDP, TR, MS, RZ, JMB, OG, and SG critically reviewed the draft. All authors read and approved the final version.

## Conflict of Interest Statement

The authors declare that the research was conducted in the absence of any commercial or financial relationships that could be construed as a potential conflict of interest.

## References

[B1] Antoniou A. S. Polychroni F. Vlachakis A. N. (2006). Gender and age differences in occupational stress and professional burnout between primary and high-school teachers in Greece. *J. Manag. Psychol.* 21 682–690. 10.1108/02683940610690213

[B2] Arbuckle J. L. (2012a). *IBM SPSS AMOS 21 User’s Guide.* Chicago, IL: IBM Corporation.

[B3] Arbuckle J. L. (2012b). *Amos (Version 22.0.0) [Computer Program].* Chicago, IL: IBM Corporation.

[B4] Arvidsson I. Håkansson C. Karlson B. Björk J. Persson R. (2016). Burnout among swedish school teachers–a cross-sectional analysis. *BMC Public Health* 16:823. 10.1186/s12889-016-3498-7 27539073 PMC4991104

[B5] Benevene P. Ittan M. M. Cortini M. (2018). Self-Esteem and happiness as predictors of school teachers’ health: the mediating role of job satisfaction. *Front. Psychol.* 9:933. 10.3389/fpsyg.2018.00933 30065670 PMC6056906

[B6] Bentler P. M. (1990). Comparative fit indexes in structural models. *Psychol. Bull.* 107 238–246. 10.1037/0033-2909.107.2.238 2320703

[B7] Boles J. S. Dean D. H. Ricks J. M. Short J. C. Wang G. (2000). The dimensionality of the maslach burnout inventory across small business owners and educators. *J. Vocat. Behav.* 56 12–34. 10.1006/jvbe.1999.1689

[B8] Brouwers A. Tomic W. Boluijt H. (2011). Job demands, job control, social support and self-efficacy beliefs as determinants of burnout among physical education teachers. *Eur. J. Psychol.* 7 17–39. 10.5964/ejop.v7i1.103

[B9] Byrne B. M. (1991). The maslach burnout inventory: validating factorial structure and invariance across intermediate, secondary and university educators. *Multivariate Behav. Res.* 26 583–605. 10.1207/s15327906mbr2604_2 26751023

[B10] Byrne B. M. (1994). Burnout: testing for the validity, replication, and invariance of causal structure across elementary, intermediate, and secondary teachers. *Amr. Educ. Res. J.* 31 645–673. 10.3102/00028312031003645

[B11] Chang M. L. (2009). An appraisal perspective of teacher burnout: examining the emotional work of teachers. *Educ. Psychol. Rev.* 21 193–218. 10.1007/s10648-009-9106-y

[B12] Cherniss C. (1980). *Staff Burnout: Job Stress in the Human Services.* Beverly Hills, CA: Sage Publications.

[B13] De Stasio S. Fiorilli C. Benevene P. Uusitalo-Malmivaara L. Di Chiacchio C. (2017). Burnout in special needs teachers at kindergarten and primary school: investigating the role of personal resources and work well-being, *Psychol. Sch.* 54 472–486. 10.1002/pits.22013

[B14] Field A. (2009). *Discovering Statistics Using SPSS*, 3rd Edn. London: Sage Publications Ltd.

[B15] Freudenberger H. J. (1974). Staff burn-out. *J. Soc. Issues* 30 159–165. 10.1111/j.1540-4560.1974.tb00706.x

[B16] George D. Mallery P. (2011). *IBM SPSS Statistics 19 Step by Step: A Simple Guide and Reference*, 12th Edn. London: Pearson Higher Education.

[B17] Gold Y. (1984). The factorial validity of the maslach burnout inventory in a sample of California elementary and junior high school classroom teachers. *Educ. Psychol. Meas.* 44 1009–1016. 10.1177/0013164484444024

[B18] Golembiewski R. T. Munzenrider R. Stevenson J. G. (1986). *Stress in Organizations: Toward a Phase Model of Burnout.* New York, NY: Praeger.

[B19] Hakanen J. J. Bakker A. B. Schaufeli W. B. (2006). Burnout and work engagement among teachers. *J. Sch. Psychol.* 43 495–513. 10.1016/j.jsp.2005.11.001

[B20] Hinkle D. E. Wiersma W. Jurs S. G. (2003). *Applied Statistics for the Behavioral Sciences*, 5th Edn. Boston, MA: Houghton Mifflin Company.

[B21] Hu L. Bentler P. (1995). “Evaluating model fit,” in *Structural Equation Modeling. Concepts, Issues, and Applications*, ed. Hoyle R. H. (London: Sage), 76–99.

[B22] Hu L.-t. Bentler P. M. (1999). Cutoff criteria for fit indexes in covariance structure analysis: conventional criteria versus new alternatives. *Struct. Equ. Modeling* 6 1–55. 10.1080/10705519909540118

[B23] Iwanicki E. F. Schwab R. L. (1981). A cross-validational study of the maslach burnout inventory. *Educ. Psychol. Meas.* 41 1167–1174. 10.1177/001316448104100425

[B24] Kantas A. Vassilaki E. (1996). Burnout in Greek teachers: main findings and validity of the maslach burnout inventory. *Work Stress* 11 94–100. 10.1080/02678379708256826

[B25] Kokkinos C. M. (2006). Factor structure and psychometric properties of the maslach burnout inventory-educators survey among elementary and secondary school teachers in Cyprus. *Stress Health* 22 25–33. 10.1002/smi.1079 28066969

[B26] Koustelios A. (2001). Personal characteristics and job satisfaction of Greek teachers. *Int. J. Educ. Manage.* 15 354–358. 10.1108/EUM0000000005931

[B27] Koustelios A. Tsigilis N. (2005). Relationship between burnout and job satisfaction among physical education teachers: a multivariate approach. *Euro. Phys. Educ. Rev.* 11 189–203. 10.1177/1356336X05052896

[B28] Liu S. Onwuegbuzie A. J. (2012). Chinese teachers’ work stress and their turnover intention. *Int. J. Educ. Res.* 53 160–170. 10.1016/j.ijer.2012.03.006

[B29] Maslach C. (1982). *Burnout, the Cost of Caring.* Englewood Cliffs, NJ: Prentice Hall.

[B30] Maslach C. Jackson S. (1986). *Maslach Burnout Inventory Manual.* Palo Alto, CA: Consulting Psychologists Press.

[B31] Maslach C. Jackson S. E. (1985). The role of sex and family variables in burnout. *Sex Roles* 72 837–851. 10.1007/BF00287876

[B32] Maslach C. Schaufeli W. B. (1993). “Historical and conceptual development of burnout,” in *Professional Burnout: Recent Developments in Theory and Research*, eds Schaufeli W. B. Maslach C. Marek T. (Washington, DC: Taylor and Francis), 1–16.

[B33] Maslach C. Jackson S. E. Schwab R. L. (1996). “Maslach burnout inventory-educators survey (mbi-es),” in *MBI Study*, 3rd Edn, eds Maslach C. Jackson S. E. Leiter M. P. (Palo Alto, CA: Consulting Psychologists Press).

[B34] Maslach C. Schaufeli W. B. Leiter M. P. (2001). Job burnout. *Annu. Rev. Psychol.* 52 397–422. 10.1146/annurev.psych.52.1.397 11148311

[B35] Montgomery C. Rupp A. A. (2005). Meta-analysis for exploring the diversity causes and effects of stress in teachers. *Can. J. Educ.* 28 458–486. 10.2307/4126479

[B36] Nunnally J. C. (1978). *Psychometric Theory*, 2nd Edn. New York, NY: McGraw-Hill.

[B37] Schaufeli W. B. Daamen J. R. H. Mierlo J. A. J. (1994). Burnout among dutch teachers: an MBI validity study. *Educ. Psychol. Meas.* 54 803–812. 10.1177/0013164494054003027

[B38] Schumacker R. E. Lomax R. G. (2004). *A Beginner’s Guide to Structural Equation Modeling*, 2nd Edn. Mahwah, NJ: Lawrence Erlbaum Associates Publishers.

[B39] Sinott E. C. Afonso M. R. Ribeiro J. A. B. Farias G. O. (2014). Síndrome de Burnout: um estudo com professores de Educação Física. *Movimento* 20 519–539.

[B40] Skaalvik E. M. Skaalvik S. (2011). Teacher job satisfaction and motivation to leave the teaching profession: relations with school context, feeling of belonging, and emotional exhaustion. *Teach. Teach. Educ.* 27 1029–1038. 10.1016/j.tate.2011.04.001

[B41] Spittle M. Kremer P. Sullivan S. (2015). Burnout in secondary school physical education teaching. *Ser. Phys. Educ. Sport* 13 33–43.

[B42] Steiger J. H. (2000). Point estimation, hypothesis testing, and interval estimation using the RMSEA: some comments and a reply to Hayduk and Glaser. *Struct. Equ. Modeling* 7 149–162. 10.1207/S15328007SEM0702_1

[B43] Stoeber J. Rennert D. (2008). Perfectionism in school teachers: relations with stress appraisals, coping styles, and burnout. *Anxiety Stress Coping* 21 37–53. 10.1080/10615800701742461 18027123

[B44] Tabachnick B. G. Fidell L. S. (2013). *Using Multivariate Statistics*, 6th Edn. Boston, MA: Pearson.

[B45] Tsigilis N. Koustelios A. Togia A. (2004). Multivariate relationship and discriminant validity between job satisfaction and burnout. *J. Manage. Psychol.* 19 666–675.

[B46] Tsigilis N. Zournatzi E. Koustelios A. (2011). Burnout among physical education teachers in primary and secondary schools. *Int. J. Humanit. Soc. Sci.* 1 53–58.

[B47] Van Horn J. E. Schaufeli W. B. Greenglass E. S. Burke R. J. (1997). A Canadian-Dutch comparison of teachers’ burnout. *Psychol. Rep.* 81 371–382. 10.2466/pr0.1997.81.2.371 9354087

